# Situational analysis of facilitators and barriers to availability and utilization of magnesium sulfate for eclampsia and severe preeclampsia in the public health system in Brazil

**DOI:** 10.1186/s12884-016-1055-0

**Published:** 2016-08-30

**Authors:** Fátima Aparecida Lotufo, Mary Angela Parpinelli, Maria José Osis, Fernanda Garanhani Surita, Maria Laura Costa, José Guilherme Cecatti

**Affiliations:** 1 Department of Obstetrics and Gynecology of the University of Campinas School of Medicine, Campinas, Brazil; 2 Sociologist and Full Professor of the Postgraduate Program on Obstetrics and Gynecology, University of Campinas School of Medicine, Campinas, Brazil

**Keywords:** Magnesium sulfate, Eclampsia, Severe preeclampsia, Quality of care, Cause-effect diagram

## Abstract

**Background:**

Eclampsia is the main cause of maternal death in Brazil. Magnesium sulfate is the drug of choice for seizure prevention and control in the management of severe preeclampsia and eclampsia. Despite scientific evidence demonstrating its effectiveness and safety, there have been delays in managing hypertensive disorders, including timely access to magnesium sulfate. To conduct a general situational analysis on availability and use of magnesium sulfate for severe preeclampsia and eclampsia in the public health system.

**Method:**

A situational analysis was conducted with two components: a documental analysis on information available at the official websites on the policy, regulation and availability of the medication, plus a cross sectional study with field analysis and interviews with local managers of public obstetric health services in Campinas, in the southeast of Brazil. We used the fishbone cause and effect diagram to organize study components. Interviews with managers were held during field observations using specific questionnaires.

**Results:**

There was no access to magnesium sulfate in primary care facilities, obstetric care was excluded from urgency services and clinical protocols for professional guidance on the adequate use of magnesium sulfate were lacking in the emergency mobile care service. Magnesium sulfate is currently only administered in referral maternity hospitals.

**Conclusion:**

The lack of processes that promote the integration between urgency/emergency care and specialized obstetric care possibly favors the untimely use of magnesium sulfate and contributes to the high maternal morbidity/mortality rates.

## Background

According to the World Health Organization (WHO), about 830 women worldwide die every day from pregnancy-related or childbirth-related complications. Virtually all deaths occur in under-resourced locations, most commonly among adolescents under 15 years of age and women living in rural areas in poor communities [1, 2]. The risk of dying from maternal causes is 33 times higher in a low-income or middle-income country than in a high-income country [2].

In a recent multicenter study conducted in Brazil, during a one-year period, 82,388 hospitalized women were monitored for any pregnancy-related complication in 27 referral maternity hospitals. Altogether these maternity hospitals represented the five regions of the country. Among the 9,555 women identified with severe maternal complications, hypertensive disorders was the main cause of hospital admission (73 %), and eclampsia was the major cause of death in the country [3, 4].

Adequate management of obstetric complications at different levels of women’s healthcare has been pointed out as the main factor for improvement in maternal and perinatal prognosis. It reinforces the concept that delay during obstetric care is the determining factor for worse outcome [5]. Currently, the drug of choice for seizure prevention and control is magnesium sulfate and the delay in using it can compromise effective obstetric care [6, 7].

In 1995, an international multicenter randomized controlled trial, The Eclampsia Trial, compared magnesium sulfate to the anticonvulsants diazepam and phenytoin. Magnesium sulfate was considered the best medication for the control and recurrence of eclamptic seizures. Women were randomized to use diazepam or phenytoin with magnesium sulfate. In both groups of women, magnesium sulfate was capable of reducing seizure recurrence in 52 and 67 %, respectively [8]. Another trial with the same characteristics, the Magpie Trial, evaluated 4.999 women with severe preeclampsia who were randomized for medication use. Magnesium sulfate decreased eclampsia by more than 50 % and major adverse events were rare, such as respiratory depression (0.9 %), respiratory arrest (0.1 %) and need for calcium gluconate (0.3 %) [9].

In a recent literature review, it was estimated that altered patellar reflex, respiratory depression and calcium gluconate requirement occurred in 1.6, 1.3 and 0.15 % of cases, respectively, during use of magnesium sulfate in 9,556 women enrolled into the study [10]. Two other studies also compared the safety of magnesium sulfate with placebo or no other anticonvulsant. The first study evaluated 11,444 women with preeclampsia and described that major adverse effects such as the absence or decrease in reflex reactivity and respiratory depression were rare occurring in 1 % of women [11]. The second study reviewed 143 publications on 10,795 women with preeclampsia and found no association between medication use and maternal death, or cardiac/respiratory arrest [12].

The drug has a very low toxicity, low cost and is simple to use by trained physicians and nurses. Drug administration may be controlled by clinical signs (diuresis, respiratory rate and tendon reflexes), and serum magnesium level measurements are not routinely needed. Toxicity is easily identified and reversed by interrupting drug use or administering the antidote, calcium gluconate [9]. Despite evidence, however, its use is limited and justified by the lack of availability, conditions of applicability, infrastructure and training for timely and proper drug use [13]. Scientific uncertainties also contribute to its underutilization, such as the mechanism of action of the antiepileptic drug, minimum effective dose [14, 15] and level of care recommended for disease management (primary, secondary and tertiary). Nevertheless, its use may be implemented in primary healthcare units (PHU) [7, 16].

Magnesium sulfate, through the decades has contributed significantly to the reduction in maternal and perinatal morbidity and mortality due to preeclampsia and eclampsia [7, 17]. There is however some clinical conditions that could still benefit from a timely use of this drug, with a general feeling that its potential impact on preeclampsia and eclampsia is not enough explored. Therefore, the purpose of this study was to conduct a general situational analysis on availability and use of magnesium sulfate for severe preeclampsia and eclampsia in the public health system.

## Methods

This was a study using a situational analysis approach [18] focusing the use of magnesium sulfate in severe preeclampsia and eclampsia in the public health system in Brazil. This was performed using two strategies: first, a documental analysis for identifying the available current pertinent national policies and legislation, and second, a cross sectional component exploring aspects on the use of magnesium sulfate at all levels of the health system. The situational analysis is known as a strategy to be implemented to gather a general overview of the current situation in a specific setting and with the purpose of further implement actions for improvement. Theoretically it involves “to define the nature and extent of the problem in the local context; to map the perceptions and experiences of key stakeholders in relation to the problem; to identify existing strategies and activities which address the problem; to identify the actors and organizations that are already active in the area; to identify the actors and organizations that could be important partners; and to identify gaps in existing strategies and activities” [18]. All these activities were judged to perfectly fit the needs for a comprehensive understanding of the problem identified.

To understand all the processes required for the drug use, we used the Fishbone or cause/effect diagram. This diagram was conceived as a tool for quality control by Kaoru Ishikawa in 1982. It was developed to identify the root cause of the problem and process restrictions, contributing to continuous quality improvement through collaborative team work. The structure of the diagram may be classified according to the convergent reasons for the problem (method, material, manual labor, machine, measurement and environment). It is presented in a graphic and synthesized manner for better visualization [19, 20].

We constructed a diagram with access to magnesium sulfate as the problem, in an attempt to understand and identify all processes involved ranging from public policies to drug use. Other studies on preeclampsia/eclampsia have already employed this method in the literature [21–23].

**Fig. 1:**
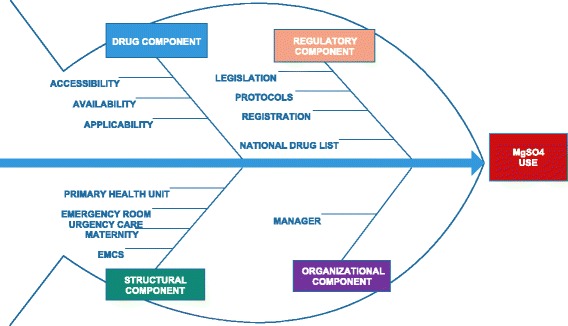
Framework with the fishbone diagram to assess to magnesium sulfate use in severe preeclampsia and eclampsia. (Edraw Max software). Structural component: EMCS (Emergency Mobile Care Service), Maternity, Emergency Room, Urgency Care, Primary Health Unit. Organizational component: manager; Pharmaceutical component: accessibility, availability, applicability; Regulatory component: legislation, protocols, registration, national drug list

Four components were identified as integrating and interrelated parts in this process, and they were classified as: a. Regulatory and governmental component (legislation, protocols, drug selection and registration); b. Drug component (accessibility, availability and applicability of the drug); c. Structural component of the healthcare network (PHU, urgency care units (UC) /emergency room (ER) and emergency mobile care service and referral maternities, according to the municipal organization); d. Organizational component (unit managers) (Fig. 1).

There were three distinct steps for the development of the method proposed: 1) documental analysis at three governmental levels; 2) exploratory analysis composed of field observation in women’s healthcare units; and 3) interview with local unit managers at each setting of the healthcare network (HCN).

For the first step, the documental analysis on regulatory/governmental legislation and public policies was performed by searching Web pages to identify public policies on women’s healthcare promoting strategic programmatic actions to reduce maternal mortality and treat women with severe hypertensive disorders during pregnancy and post-partum period. This search was performed at the three correspondent government levels: federal, state and municipal, considering that some differences could exist among them taking into account their relative autonomy [24]. Websites accessed were from the Ministry of Health (MH), the São Paulo State Department of Health (SDH) and the Campinas Municipal Department of Health (MDH).

In the main webpage of the Ministry of Health, we accessed the National Health System (SUS) legislations to identify those providing information on the country’s health system that were relevant to the study. We sought actions, programs and strategies exploring rules of the Department of Basic Care, Family Health Program, Primary Healthcare, Stork Network and Urgency/Emergency Network.

Still as part of documental analysis, we found protocols containing recommended treatment for severe hypertensive disorders during pregnancy and postpartum period. We searched for protocols in the Ministry of Health online library, as well as in the São Paulo State Department of Health and the Campinas Municipal Department of Health.

For analysis of the regulatory component of the drug, we checked its record and inclusion in the drug lists (national, state and municipal). Its characteristics for indication and prescription were taken into account, following the National Sanitary Surveillance Agency (ANVISA), the organ responsible for drug registration and approval in the country.

The second and third steps were regarded exploratory analysis from field observation in women’s healthcare units and interview of managers. Assessment of availability, accessibility and applicability of magnesium sulfate was conducted by field observations and simultaneous interviews with managers in PHU, UC, ER, EMCS and referral maternity hospitals. In order to avoid any courtesy bias and to assure homogeneity, the visits were performed without previous scheduling by only one interviewer who is from the research team (FAL). Interviews used a questionnaire specifically built for the study applied from July to December 2015. It contained information on availability of technical protocols for management of the condition in all health care levels, the flow of the woman in situations of hypertensive urgency and emergency, equipment, human and material resources needed by the level of care.

**Fig. 2:**
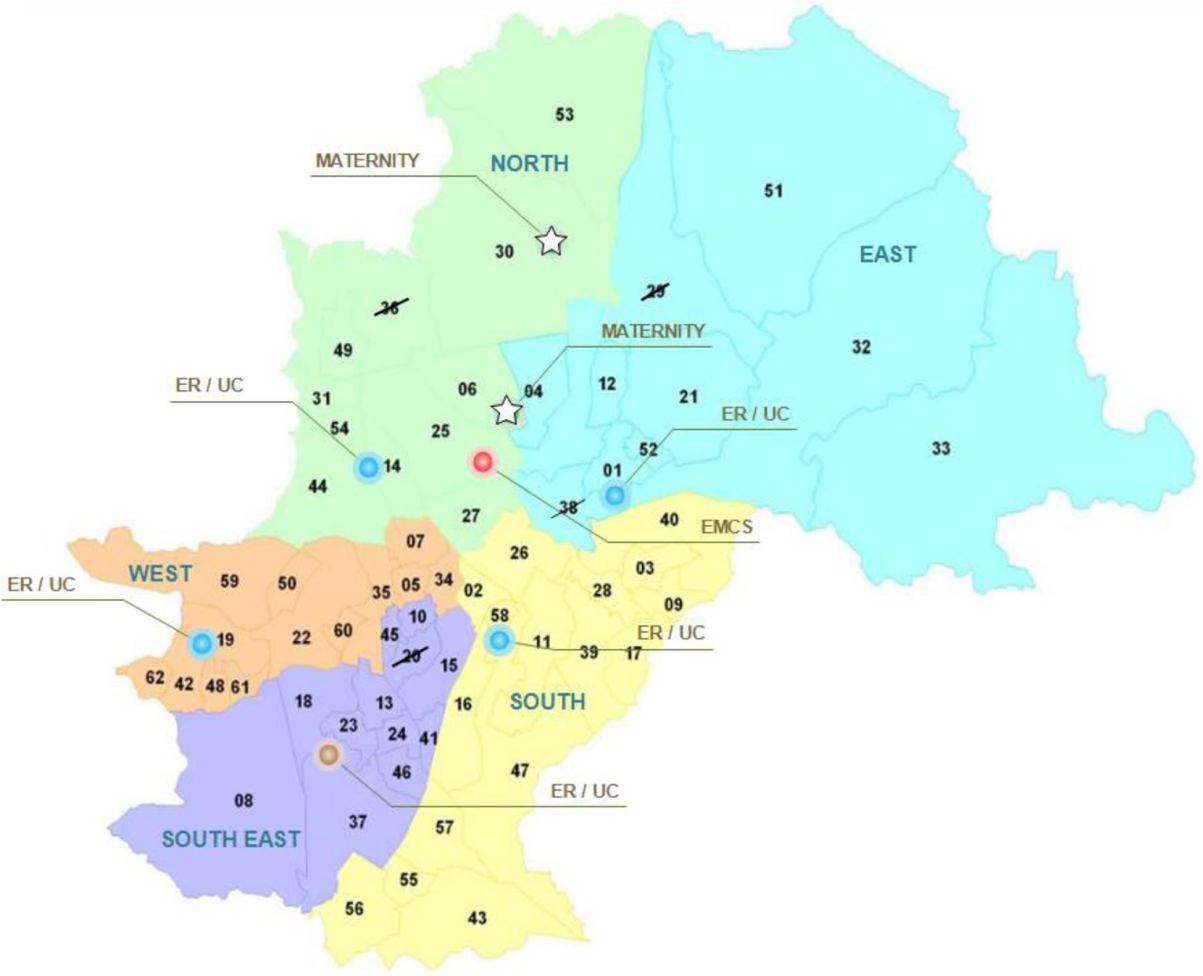
Healthcare Network (Health facilities) of the municipality of Campinas, SP, Brazil. Arabic numerals: geographic locations of the PHU (primary health unit); ☆ : Maternity hospitals; ○: UC/ER (urgency care/emergency room). crossed out numbers: non-participating PHU

For the structural component of the study a case study was performed with the municipality of Campinas using a cross sectional design. We selected a convenience sample from Campinas, including its whole health units at all levels, the Health Care Network (HCN). It contains 63 PHC, 4 UC, 2 ER, the EMCS and 3 referral maternity hospitals (Fig. 2) [25]. Campinas is the third largest city in the State of São Paulo, in the southeastern region of Brazil. It has 1.080.034 inhabitants and 504,175 women [26]. The city is divided into five districts (north, south, east, southeast and northwest) with around 200.000 inhabitants in each one, which has also its district healthcare surveillance and referral hospital units according to neighborhood and complexity.

The data collected were entered in Excel 2007 program spreadsheets. Database was analyzed for logical consistency and any errors identified were corrected. The absolute (n) and relative (%) frequencies of categorical variables were calculated. To construct the diagram with its layout, the “Edraw Max” software was used.

The study protocol was approved by the Institutional Review Board (IRB) from the School of Medical Sciences of the University of Campinas, by the Research Ethics Committee of MDH and by the Clinical Directors of maternity hospitals. A written free informed consent term was used for managers and applied during the interviews (IRB protocol number 658.325).

## Results

Our results are presented according to the four components of the fishbone diagram and are available in the summary table (Table 1):

**Table 1:** Magnesium sulfate in preeclampsia/eclampsia in public health system in the municipality of Campinas, SP - Brazil

Component	Facilitator	Barriers
Regulatory		
Legislation	✓ Healthcare Pact of 2006 - administrative rule n° 399/MH	✓ Application of MgSO_4_ is made only in hospitals ✓ Level for application of MgSO_4_ is not available
	✓ Stork Network of 2011 – Administrative rule n° 1459/ MH	
		✓ MgSO_4_ 20 and 50 % is included in the basic and essential list of drugs for hospital units
	✓ Urgency/ Emergency Network - Administrative rule n° 1600/ MH	
		✓ Prioritizes trauma, cardiovascular and cerebrovascular care
Registration	✓ RENAME – MH and SDH-SP: has MgSO_4_,	✓ No specification for use in hypertensive syndromes
	✓ Drug registration - ANVISA	
		✓ Municipal list: no reference to MgSO_4_
Protocol	✓ Urgency/emergency protocol /2000 - MH	✓ Municipal Protocol: lacking
		✓ Level for application of MgSO4 is not available
	✓ High-risk pregnancy protocol /2012 – MH	
		✓ No specification for use in hypertensive disorders
	✓ SDH-SP protocol / PN and Puerperal Technical Manual 2010	
	✓ Basic Life Support (BLS) and Advanced Life Support (ALS) / MH	
	✓ EMCS municipal protocol /guidance of patient transportation in cases of pregnancy-related hypertension	
Drug		
Conditions of applicability	✓ All	✓ PHU: only essential equipment
Availability	✓ Presence UC/ ER and Maternity Hospitals	✓ Lacking PHU and EMCS
Structure		
Equipment	✓ Available at UC, EMCS and Maternity Hospitals	✓ PHU: only essential equipment
Physical	✓ Distribution apparently adequate for HCN	
	✓ PHU with medication room adapted to local architecture	
	✓ UC, EMCS and Maternity Hospital with adequate architecture	
Organizational	✓ Presence of physicians	✓ PHU: destined to ambulatory scheduling
		✓ Insufficient number of physicians for urgencies in PHU
		✓ Exclusion of UC/ER in the care of pregnant women
		✓ Protocol lacking in UC/ER, available in 56 % of PHU, present in the maternity hospitals
		✓ Exclusive use by the obstetrician
		✓ Responsibility not established
		✓ Elective work activity (programmed schedule)
		✓ Lack of training

*ANVISA* National Sanitary Surveillance Agency, *EMCS* Emergency Mobile Care Service, *ER* emergency room, *HCN* Healthcare Network, *MH* Ministry of Health, *PHU* primary health unit, *PN* prenatal care, *RENAME* National Drug List, *SDH* State Department of Health, *UC* urgent care

### Regulatory/governmental component

This component corresponds to the documental analysis as planned. In the Ministry of Health website on basic legislation for the National Health System, it was possible to identify specific legislation on the reduction of maternal mortality and management of hypertensive disorders. Administrative rule GM number 399 of February 22^nd^, 2006 initiated the Health Pact, restructuring the health system in the country into primary healthcare (PHC) and healthcare networks (HCN). It prioritized the consolidation and qualification of Family Strategy as the model of PHC for modelling the HCN [27]. One of its goals is to reduce maternal mortality in the country, ensuring “materials and medication for treatment of Hypertensive Syndromes in childbirth”. The HCN organizes healthcare services into maternal healthcare, infant healthcare, disability healthcare, urgent and emergency care, among others [28].

The Stork Network, instituted within the scope of the National Public Health System (SUS) under the administrative rule 1459 of June 24^th^, 2011, organizes maternal healthcare. It determines the healthcare settings a woman will follow in her itinerary. The aim of the Stork Network is to ensure that the woman receives humanized care during pregnancy, childbirth and the postpartum period. It is focused on childbirth care, offering a guarantee of access, care and resolution [29]. All healthcare settings are equally relevant and distinguished by technological density. PHC is responsible for the interrelationship and maintenance of these settings, organizing different tasks in different settings to overcome system fragmentation [28].

To instruct regional and municipal actions on Stork Network implementation, administrative rule 650 of November 5^th^, 2011 technically regulates functioning of obstetric and neonatal healthcare services [30]. In this resolution, injectable magnesium sulfate at concentrations of 20 and 50 % is included in the basic and essential list of drugs for hospital units [31].

Each healthcare setting shall fulfill specific aims in urgent care. The primary health unit will be responsible for initial medical care until patient referral to another healthcare setting. The EMCS will render the care required during transportation. The UC unit is responsible for the resolution and qualified care of acute clinical cases. It delivers initial medical care in surgical cases or trauma, stabilizing patients and performing early diagnostic investigation, defining urgency destination and hospital referral. However, the urgency/emergency network prioritizes cardiovascular and cerebrovascular care, trauma, and does not include maternal care in this priority [32].

To assist pregnant women diagnosed with severe preeclampsia and eclampsia, standardized clinical protocols on the timely and proper use of magnesium sulfate are required in a hospital and non-hospital setting. The Ministry of Health has a protocol on Maternal Urgencies and Emergencies [33] and a Technical Manual on High-Risk Pregnancy [34], where there are guidelines for the diagnosis and treatment of severe preeclampsia/eclampsia and for the timely and proper use of magnesium sulfate. The Sao Paulo State Department of Health also has a Technical Manual of Prenatal and Puerperal Care [35]. However, none of the protocols refers to the setting where the medication should be applied.

The EMCS has two clinical protocols available: Basic Life Support and Advanced Life Support. None of these protocols contains recommendations for the use of magnesium sulfate [36]. The EMCS Manual of Medical Routine of the MDH Campinas predicts transportation and approach of the pregnant woman with severe hypertensive disorders, although it does not specify the use of magnesium sulfate [37].

Availability of magnesium sulfate was confirmed by the drug registration in the National Sanitary Surveillance Agency (ANVISA), in concentration of 10 and 50 % for parenteral use [38] and included in the National Drug List (RENAME), without specifying its use in Obstetrics [39]. At the website of the Sao Paulo State Department of Health, we found the same medication list as in the Ministry of Health (RENAME). The MDH of Campinas has a standardized drug list to supply healthcare units that does not include magnesium sulfate in any of its injectable presentations [40, 41].

### Drug component

Access to medication is dependent on several processes, starting at entry into the PHU and family healthcare program. The municipality also has good access to prenatal and childbirth care; 12,573 or 78.7 % of pregnant women had seven prenatal visits or more in 2014 and 99.6 % of deliveries in the metropolitan region of Campinas occurred in hospitals [42, 43]. Quality assessment of prenatal care according to risk identification, early diagnosis of preeclampsia/eclampsia and early treatment of severe cases were not aims of this study. However, the medication was not part of the PHU drug list and was not available for that purpose. On the other hand, in our field observation we also identified minimum conditions for safe drug use (Table 2).

**Table 2:** Available resources for management of magnesium sulfate at health facilities from the Healthcare Network in Campinas, SP, Brazil

Field Observation	PHU		UC + ER		EMCS		MATERNITY	
	n (59)	%	n (5)	%	n (1)	%	n (2)	%
Protocol for SPE/E Diagnosis and Treatment	33	56	0	0	0	0	2	100
Minimum conditions for intravenous use^a^	59	100	5	100	1	100	2	100
MgSO4, 10 % vials	0	0	5	100	0	0	2	100
MgSO4, 50 % vials	0	0	0	0	0	0	2	100
Calcium gluconate, 10 % vials	52	88	5	100	1	100	2	100
Cardiac monitor	8	14	5	100	1	100	2	100
Pulse oximetry	34	58	5	100	1	100	2	100
Cardiac defibrillator	9	15	5	100	1	100	2	100
Laryngoscope	57	97	5	100	1	100	2	100
Orotracheal cannula	59	100	5	100	1	100	2	100

*EMCS* Emergency Mobile Care Service, *ER* emergency room, *PHU* primary health unit, *UC* urgent care

^a^Minimal conditions for intravenous MgS04 use: syringe, needle, tubing line, fluid, adhesive tape and cotton

### Structural component

In terms of health service structure, it was possible to visualize three action groups and services institutionally grouped under the National Health System: primary care, formed by family strategy and PHU; medium- and high-complexity care formed by outpatient facilities; and hospital admissions in public maternity hospitals.

Each primary healthcare unit is responsible for the care of approximately 20,000 inhabitants. Healthcare is delivered by multiprofessional teams. Families are enrolled and healthcare is provided by scheduled visits. Along with primary health units, family healthcare programs are aimed to expand and qualify basic care, improving its resolution. Both units are part of the “Integral Urgency/Emergency Care Plan”. Attributions of these units include patient reception and assessment of the risk and complexity of acute urgent case arriving spontaneously [44].

All PHU visited (*n* = 59/63) had a medication room and nursing staff for management and surveillance during working hours. In the medication room, gurneys, armchairs, IV lines, sphygmomanometers and stethoscopes were identified, although not all settings had the physical structure to care for urgent cases. Some PHU had greater surveillance resources, with devices such as pulse oximetry, cardiac monitor and defibrillator in 34 (57.6 %), 8 (11.6 %) and 9 (15.9 %) units, respectively. Clinical protocols were irregularly available at various maternal care units (PHU 33/59; UC + ER 0/5; EMCS 0/1; maternity 2/2). (Table 2).

UC and ER have the infrastructure, materials, equipment and teams prepared for urgency/emergency care due to the free demand of critical patients. These facilities function on a 24-h basis and ensure clinical support until patient transfer to a hospital. However, these units have no obstetrician and/or neonatologist and/or specific beds in its work structure.

EMCS has the resources for intensive care and ensures adequate transportation of clinical, pediatric and obstetric urgent cases to referral centers, in addition to all other situations that require removal to a more complex level of care.

Three maternity hospitals, two of them visited, account for urgency/emergency care in pregnancy, childbirth and postpartum period, both with the structure to use magnesium sulfate and deliver adequate maternal-infant care. Healthcare is provided by the medical staff, nursing staff, multidisciplinary teams and other support medical specialties, in a 24-h daily.

### Organizational component

During visits, it was possible to observe the presence of physicians, nursing staff and multidisciplinary healthcare team in the facilities. We were able to interview healthcare unit managers, inquiring about magnesium sulfate, drug availability and maintenance, along with experience in hypertensive pregnancy-related urgency.

Reports of all PHU managers, i.e. 100 % visited, reiterated that the physical structure and technical resources were limited for magnesium sulfate administration. In addition, they did not participate in the choice of drugs standardized by the MDH, according to complexity of care. In these units, managers questioned which professional would be apt for drug use, since it was known that “the drug was exclusively used by obstetricians and gynecologists in a hospital setting”. Difficulties in receiving the urgent case were also reported, since work is performed according to a programmed schedule. The number of physicians is inadequate to care for this demand without interruption of elective care. Time is restricted for case management, since the unit is not open full-time. Nevertheless, there was a high demand for proper training in magnesium sulfate use in preeclampsia and eclampsia.

In the UC and ER settings, it could be observed that technical conditions and infrastructure permit the provision of more complex care. However, local managers reported that these units have no access to pregnant women, since they are instructed to seek maternity hospitals in case of urgency and emergency. The reason is that there is no obstetrician and/or medical team available for patient care.

In the EMCS, the manager reported that the healthcare team was prepared and motivated for the use of magnesium sulfate in preeclampsia and eclampsia. It was believed that these practitioners could contribute to improved maternal clinical conditions during more prolonged transportations. Maternity managers described that clinical conditions of the transferred women could have been better, if magnesium sulfate had been administered during the entire healthcare period until patient referral. Their report confirms the statement made by the EMCS manager.

## Discussion

Analyzing the components of the fishbone diagram, our study identified barriers to the timely proper use of magnesium sulfate for severe preeclampsia in the national public health system (SUS) in the city of Campinas. Magnesium sulfate registration for use in the country, its selection in the national drug list, the presence of specific protocols, and maternal healthcare in the municipality of Campinas with many PHU strategically distributed, according to regional population density, would allow the reception and management of preeclampsia and eclampsia, complying with legislation and working SUS guidelines. However, in the daily unit routine, many barriers still need to be overcome.

Government understanding that preeclampsia and eclampsia are public health issues with participation in maternal death, guides legislative strategies towards dealing with the problem. Regulatory national policies (primary healthcare, the Stork network and urgency/emergency care) include maternal hypertensive urgency care. However, these policies are not interlinked and articulated to ensure integrated and continuous maternal healthcare. A clear example of this inadequacy is the official recognition that magnesium sulfate is only obligatory in hospital settings, with potential loss of opportunities do timely and proper management of severe clinical situations. Studies have already demonstrated the use of magnesium sulfate in primary health care could reduce the recurrence of eclampsia by 78 % [14].

These governmental policies should be offered to municipalities by the Ministry of Health. However, managers have responsibility to decide whether the actions proposed will be developed or not in the municipality. Choosing not to participate in these policies may negatively influence the quality of care, contributing to increased sequelae and death during pregnancy and postpartum period [45].

Clinical protocols are quality tools in healthcare that point towards diagnosis and treatment. Guidelines, actions and resources necessary for a certain purpose are established. The elaboration of these tools connects legislation and scientific evidence to clinical practice. It ensures a safe source of knowledge of the timely and proper use of magnesium sulfate in severe preeclampsia and eclampsia. Nevertheless, to contribute to timely drug use, protocols need to be systematically present in the workplace for consultation. Strategies to understand and learn the protocol, in addition to the working organization proposed by the HCN, should be part of SUS organizational structure to construct and/or recycle knowledge of health professionals in a close, constant and effective manner [46]. Overseeing the health professional for capacitation and optimal working conditions, contributes to the good quality of the service, processes, results, strengthening relationship with the work unit [47]. In fact, the results of the current study showed that not all PHU, urgency and emergency services and mobile services have guidelines easily available for consultation, nor a systematic regular training program of personnel for using magnesium sulfate was implemented.

Our results indicated that the PHU in Campinas in its working organization understands that it is not eligible for urgent or emergency care. It recommends that pregnant women seek referral maternity hospitals in critical situations. This limits its action and compromises the level of care. Its limited technical resources and insufficient number of healthcare workers to care for urgent cases reinforces this understanding and negatively affects the capacity of health professionals to provide high-quality healthcare.

In an emergency situation, the pregnant woman is instructed to seek a maternity hospital for first medical care. Rapport is lost and the opportunity to evaluate the required complexity is missed. The pregnant woman is responsible for her transportation, often depriving her of the high-quality EMCS transport, in addition to an adequate and timely management of preeclampsia/eclampsia. On the other hand, unnecessary patient removal to a referral hospital unit increases costs and overburdens ER in maternity hospitals. The healthcare specialist is dislocated from assisting severe cases to care for cases of low complexity and simple resolution. This organizational healthcare practice may delay the proper use of magnesium sulfate, deteriorating maternal and perinatal prognosis [48].

A lack of adequate and timely transportation, lack of initiative to seek help or recognize alert signs for the diagnosis, and adequate care in the referral hospital unit are the three main causes of delays associated with maternal death. Strategies to reduce delay may contribute to decreased morbidity and mortality rates [5]. A recent systematic review identified a delay rate of 92.3, 82.1, and 63 % in the transportation, in the capacity to recognize alert signs, and in offering a quality of care, respectively, associated with a severe maternal outcome [48].

EMCS in the municipal healthcare organization plays a vital role in the management of maternal urgencies. Its healthcare workers are responsible for the safe transportation and management of maternal emergency cases. Nevertheless, the occurrence of severe preeclampsia and eclampsia is rare and widespread, providing little experience to EMCS healthcare workers. Since magnesium sulfate is not available for this specific treatment, proper drug use is delayed until the patient arrives at the hospital and care is provided by obstetricians and ICU professionals. Experience in proper use of the medication is delegated only to these healthcare professionals.

The need to organize and coordinate health care services at various levels of technological density, economic restrictions and containment of expenditure led Brazil, as well as countries from the European Union, to amplify the functions of primary healthcare. Its actions have been qualified, strengthened and expanded. This seems to be the pathway to humanized, problem-solving oriented care, committed to the rational use of resources [49]. However, the PHU will remain underused, as long as the municipality recommends that pregnant women should seek care in maternity hospitals in urgent situations, and as long as the reception of urgencies in these units is negatively perceived by pregnant women and their relatives [50].

Even with qualification and strengthening of primary healthcare, and capacitation of practitioners in the use of magnesium sulfate in severe preeclampsia and eclampsia, access to medication demands a complex construction. The drug should be available in healthcare units and prescribed by a medical practitioner, administered either by the physician who ordered the drug or by another professional. It should be dependent on resources for drug administration before benefitting the pregnant woman. Operationalization of this process passes through various actors. These actors are required to know the reality of maternal hypertension in our country. Furthermore, these actors need to be persuaded and involved in the conduction and timely and proper use of the drug. Lack of drug availability, structural and technical resources, in addition to skilled health professionals who are committed are issues that are also shared by other countries dealing with the problem [21–23].

Our study had some limitations. Data were collected at a specific time and barriers and facilitators could vary over time at each unit visited. Lack of access to medication in the PHU were barriers only mentioned by local managers and not by pregnant women or other health professionals at each unit visited, even if they were inserted in the local SUS context. Only two out of three maternities responsible for maternal care could be visited. The study was conducted in only one municipality of the southeastern region of Brazil. However, the regulatory and drug components identified in this study, are defined for the whole country.

Furthermore, considering data from a national study, the prevalence of eclampsia was about four times higher in the North, Northeast and Center West regions compared to the South and Southeast regions [4], thus it could be extrapolated in a systematic and timely manner that lack of access to magnesium sulfate is even higher in other regions in Brazil.

The fishbone diagram is a tool that was helpful in identifying barriers for the use of magnesium sulfate in severe preeclampsia and eclampsia. It allowed the study to point out the multifactorial challenges to effectively achieve the provision of high-quality care in hypertensive emergencies during pregnancy.

## Conclusions

Interventions such as the inclusion of magnesium sulfate in the national drug list, starting drug use in the PHU, specifying that it is the drug of choice for treatment of severe preeclampsia and eclampsia with specific protocols and simplified leaflets for medication use are required. In addition, refreshing courses and continuous professional training, restructuring the PHU to ensure the supply, distribution and applicability of the medication at all levels of maternal care may contribute to improved rates of maternal and perinatal morbidity and mortality.

## Abbreviations

ANVISA: National sanitary surveillance agency
EMCS: Emergency mobile care service
ER: Emergency room
HCN: Healthcare network
ICU: Intensive care unit
IRB: Institutional review board
MDH: Municipal department of health
PHC: Primary healthcare
PHU: Primary health unit
RENAME: National drug list
SDH: State department of health
SUS: National health system
UC: Urgency care
WHO: World health organization
